# Expression, characterization, and application potentiality evaluation of recombinant human-like collagen in *Pichia pastoris*

**DOI:** 10.1186/s40643-022-00606-3

**Published:** 2022-11-17

**Authors:** Lingling Ma, Xiaolin Liang, Shiqin Yu, Jingwen Zhou

**Affiliations:** 1 Science Center for Future Foods, Jiangnan University, 1800 Lihu Road, Wuxi, 214122 Jiangsu China; 2 Key Laboratory of Industrial Biotechnology, Ministry of Education and School of Biotechnology, Jiangnan University, 1800 Lihu Road, Wuxi, 214122 Jiangsu China; 3 Engineering Research Center of Ministry of Education On Food Synthetic Biotechnology, Jiangnan University, 1800 Lihu Road, Wuxi, 214122 Jiangsu China; 4 Jiangsu Province Engineering Research Center of Food Synthetic Biotechnology, Jiangnan University, Wuxi, 214122 China

**Keywords:** Recombinant human-like collagen, *Pichia pastoris*, Potential applications

## Abstract

Collagen is a biofunctional protein that has been widely used in many fields, including but not limited to biomedical, cosmetics and skin care, food, and novel materials. Recombinant collagen has great potential as an alternative to collagen extracted from animals because it avoids the immune response, and the yield and properties are stable. However, challenges remain in the industrial application of recombinant collagen, including improving the expression yield, reducing the cost of purification for industry and expanding applications. In this study, a cloning and recombination method was used to heterologously express the recombinant human-like collagen (RHLC) in *Pichia pastoris* GS115 using the pPIC9k expression vector. The RHLC expression titre was 2.33 g/L via a 5-L fermenter, and the purification was completed within 48 h and was 98% pure. The characteristics of RHLC were investigated. Furthermore, potential applications for RHLC were explored, such as basal collagen sponge preparation, forming films with chitosan and production of collagen hydrolysed peptides. RHLC has various potential applications due to its triple helical structure, thermostability, good biocompatibility and film-forming ability.

**Graphical Abstract:**

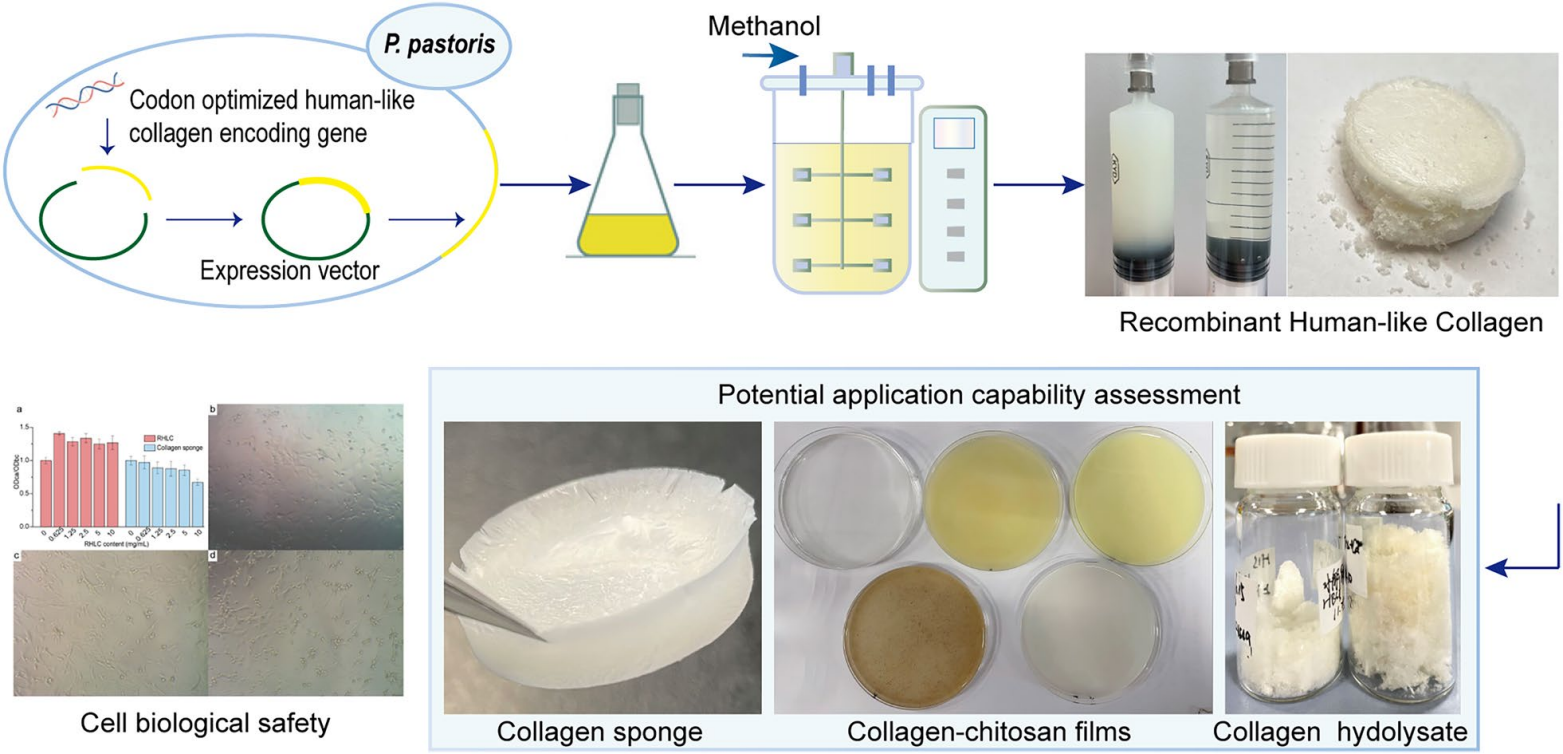

**Supplementary Information:**

The online version contains supplementary material available at 10.1186/s40643-022-00606-3.

## Introduction

Collagen is a natural biopolymer that forms important structures, such as skin, muscle, bone and blood vessels. It is widely used in many fields, including the food, pharmaceutical, cosmetics and biomedical industries. The triple helical structure is the basis of the physiological function of collagen (Fidler et al. 2018). Collagen molecules form a unique triple helical structure by self-aggregating, resulting in a filamentous and fibrous microstructure. The repeating amino acid sequence of the triple helical domain is (Gly-X-Y)n, in which Gly is glycine, X is usually proline, and Y is typically hydroxyproline (Brodsky and Persikov 2005). This sequence accounts for ~ 56% of the total protein (Sorushanova et al. 2019).

The basic repeating sequence of the triple helical region has been utilized to design and synthesize functional units for many protein materials, and collagen has been applied to many fields using this property (Asghari et al. 2017; Ebhodaghe 2021). As collagen promotes wound healing (Feng et al. 2020; He et al. 2020), biomaterials based on collagen are important in applications, such as collagen sponges, as a potential platform for drug delivery (Lin et al. 2019). Moreover, along with the development of protein expression technologies, additional recombinant collagens have been designed and expressed to develop novel materials that can be applied to various fields (Irawan et al. 2018; Zarei et al. 2021).

Collagen is prepared in many ways, but extraction from animal tissues is the current main production mode. However, the possibility of immunogenicity and contamination with pathogens are limiting factors of extracted collagen (Sorushanova et al. 2019), and religious issues and animal welfare problems also limit production from this method. Therefore, collagen must be more effectively and safely prepared using a high-yield method, with unique sequences and a stable structure. Animal cells can be used to produce collagen, but cell culture requires nutrients and it suffers from high cost and low yield, preventing it from meeting production needs (Worthen et al. 2020). Some cells of non-mammalian origin are not amenable to clinical transformation (Choi et al. 2021; Stephens et al. 2018). In addition, although the synthesizing collagens can address safety, purification and yield issues associated with extracting the protein from animals or cell production, the high cost of production made it unable to industrialize.

Microorganisms have been used to express recombinant collagen, which not only solves the problems of poor stability between batches, potential biological toxins and pathogenic residues, but also shortens the production cycle. Two microbial expression systems have been employed to produce recombinant human-like collagens based on *Escherichia coli* (Fan et al. 2002; Hou et al. 2006) and *Pichia pastoris* (Wang et al. 2014). Recombinant collagens can be expressed in a triple helical structure, although their properties are different from those of natural fibres (Ruggiero and Koch 2008). *P. pastoris* has advantages as an expression host for heterologous proteins, including high-density fermentation, sophisticated protein secretion and expression system and ease of purification. In a previous study, a water-soluble recombinant human-like collagen (RHLC) was expressed in *E. coli*, and developed for cosmetics applications (Zhang 2017). However, low production limited the further application of this RHLC. To enhance the expression yield and enlarge the application range, we chose *P. pastoris* as the expression host for human-like collagens in the present study. *P. pastoris* yeast uses methanol as the sole carbon and energy source, so utilizing the alcohol oxidase (P_AOX_) promoter efficiently expresses exogenous genes induced by methanol (Ahmad et al. 2014). More potential applications have been tentatively explored based on purified RHLC.

In this study, the RHLC gene was synthesized and the codon was optimized by the GS115 *P. pastoris* expression system. A 5-L fermenter in a shaker flask was induced with methanol. The ammonium sulphate precipitation method was used for rapid and simple purification, and we obtained large quantities of highly pure collagen by dialysis with ice-cold ultrapure water. The basic characteristics of RHLC were examined, and various applications were primarily explored, including in vitro cytotoxicity of the collagen sponge, the film-forming ability of film material and the molecular weight distribution of collagen hydrolysate.

## Materials and methods

### Strains, plasmids and culture media

The *E. coli* JM109 and *P. pastoris* GS115 strains were used to construct the recombinant plasmids and express collagen. The pPIC9k expression vector was obtained from our laboratory stock. A plasmid extraction kit and a polymerase chain reaction (PCR) purification kit were purchased from Vazyme Biotech Co., Ltd (Nanjing, China) and Thermo Fisher Scientific (Waltham, MA, USA), respectively. The restriction enzyme *Sac*I was purchased from Takara Biomedical Technology Co., Ltd. (Beijing, China). The NuPAGE™ Bis–Tris precast adhesive, NuPAGE™ LDS sample loading buffer (4 ×) and NuPAGE™ MES SDS running buffer were obtained from Invitrogen Life Technologies (Carlsbad, CA, USA). Bradford protein assay kits were purchased from Tiangen Biotech Co., Ltd (Beijing, China). The Hiload 26/600 Superdex 200 pg (prep grade) gel filtration column and AKTA Pure chromatography system were obtained from GE Healthcare Life Sciences (Tirat, Hacarmel, Israel).

The YPD culture medium contained 20 g/L glucose, 20 g/L peptone and 10 g/L yeast extract. The MD medium used to screen the histidine-defective strains contained 20 g/L glucose, 13.4 g/L YNB, 0.4 × 10^–4^ g/L biotin and 20 g/L agar. The BMMY expression-inducing medium contained 20 g/L tryptone, 10 g/L yeast extract, 3 g/L K_2_HPO_4_, 11.8 g/L KH_2_PO_4_, 13.4 g/L YNB, 0.4 × 10^–4^ g/L biotin and 0.5% (v/v) methyl alcohol. The fermentation medium contained 26.7 mL/L of 85% H_3_PO_4_, 1.2 g/L of CaSO_4_·2H_2_O, 18.5 g/L of K_2_SO_4_, 15 g/L of MgSO_4_·7H_2_O, 4.2 g/L of KOH, 40 g/L of glycerin and 4.35 mL/L of PTM1 trace salt solution. The PTM1 trace salt solution was purchased from Coolaber Technology Co., Ltd (Beijing, China), and was added to the medium before inoculation. All media were sterilized at 121 °C for 20 min. All other chemicals were of analytical grade.

### Expressing RHLC in *P. pastoris*

#### Construction of recombinant *P. pastoris*

The amino acid sequence of human-like collagen in this study refers to the hydrophilic amino acids based on type I and III human collagen (Zhang 2017). The triple-helix region consisted of eight continuous repeating units of GERGDLGPQGIAGQRGVVGERGERGERGAS. The collagen-coding gene sequence codon was optimized according to *P. pastoris*, synthesized by Genewiz Co. Ltd. (Suzhou, China) and inserted into the site pPIC9k plasmid following the α-factor secretion signal (Additional file 1).

The expression vector was linearized with *Sac*I restriction endonuclease and the purification of digestion products was integrated into *P. pastoris* GS115 competent cells by electric shock transformation. MD solid medium plates were used to screen the positive clones into which the genes were successfully integrated, and YPD solid medium plates containing 1–4 mg/mL G418 were used to screen transformants with a high gene copy number.

#### Expression of RHLC in *P. pastoris*

Small-scale RHLC production was achieved by fermentation in a 250 mL flask at 30 °C with shaking at 220 rpm. The seed solution was inoculated into YPD medium until the OD_600nm_ reached 2.0–6.0. The cells were collected after centrifugation at 5000 rpm for 15 min. Next, the cells were suspended in BMMY medium at a ratio of five times the seed solution and supplemented with 0.5% (v/v) methanol every 24 h to induce RHLC expression. The induction process lasted 96–120 h at 28 °C and 220 rpm.

Larger-scale RHLC production was achieved by fermentation in a 5-L fermenter, and methanol was used to induce expression. The seed solution was added to a sterile ionic medium with glycerol as the carbon source. When the dissolved oxygen content began to rise, the medium was fed-batch cultivated with 50% glycerol until the wet cell mass reached 180–210 g/L. The expression of RHLC was induced by the continuous flow of methanol at a rate of 6.0–7.0 mL/L at 22 °C. Protein expression was assessed by sodium dodecyl sulphate-polyacrylamide gel electrophoresis (SDS–PAGE).

#### Purification of RHLC

Cell suspensions fermented in shaker flasks were centrifuged using a low-temperature high-speed centrifuge at 4000 rpm for 30 min at 4 °C to obtain the fermentation supernatants. A concentrate of 20% ammonium sulphate was used to remove miscellaneous protein by centrifugation of the sediment, and 60% ammonium sulphate was used to precipitate the RHLC protein. The protein precipitate obtained after centrifugation was dissolved in ultrapure water, and the RHLC solution was dialyzed in ultrapure water using a dialysis bag with a molecular weight cut-off of 8–10 kDa. Finally, the dialyzed samples were centrifuged at room temperature to remove impurities, such as water-insoluble proteins and cell fragments not removed in the previous steps. The samples were freeze-dried for further study.

The freeze-dried samples were redissolved and assessed using a Hiload 26/600 Superdex 200 pg (prep grade) gel filtration column attached to the AKTA instrument. The peak area was calculated to obtain the purity of the RHLC.

### Characterization analyses

#### Circular dichroism (CD) spectroscopy

A Chirascan-plus spectrometer (Applied Photophysics, Shanghai, China) was used to record the CD spectra in the far-UV range at a wavelength of 190–260 nm. The step size was 1.0 nm, the spectral bandwidth was 2 nm and the scanning speed was 0.07 s per point. Experiments were performed in triplicate.

#### Fourier transformation infrared (FTIR) spectroscopy

The spectra of the freeze-dried collagen samples and the collagen sponge were measured using an FTIR spectrometer (NEXUS Co., Tempe, AZ, USA) in attenuated total reflection mode. An air spectrum was used for background correction, and all spectra were collected over a wavelength range of 650–4000 cm^−1^, as reported previously (Xiao et al. 2021).

#### Thermal stability analysis

The thermal stability of the freeze-dried RHLC and the collagen sponge was evaluated using differential scanning calorimetry (DSC) (Q200; TA Instruments, New Castle, DE, USA). A linear temperature program ranging from 40 to 200 °C was employed at a heating rate of 5 °C/min under nitrogen. Samples (2–4 mg) were sealed in an aluminium crucible with an empty crucible as a reference. The denaturation temperature (*T*_*d*_) was denoted as the endothermic peak temperature.

#### X-ray diffraction (XRD)

XRD patterns were collected using a Bruker D8 Advance X-ray diffractometer (Bruker AXS, Karlsruhe, Germany) at a 40 kV tube voltage and a 40 mA tube current. The scanning range 2θ was from 5° to 80° with a scan speed of 5° per min.

#### Scanning electron microscopy (SEM)

The structure and surface morphology of the freeze-dried collagen samples was examined using an FEI QUANTA 200 scanning electron microscope (Thermo Fisher Scientific). The samples were coated with platinum and observed at an accelerating voltage of 5.0 kV.

### Potential applications of RHLC

#### Basal collagen sponge preparation and in vitro cytotoxicity evaluation

The collagen sponge was prepared rapidly using a high-concentration collagen solution. Briefly, the high-concentration (20–25 mg/mL) collagen solution was poured into a mould after dialysis, incubated overnight on ice to form a gelatinous state and freeze-dried to obtain the collagen sponge. The characteristics of the collagen sponge were examined as described in “Characterization analyses” section.

An in vitro cytotoxicity evaluation was performed using c2c12 mouse muscle stem cells during their logarithmic growth phase (cell density 10 × 10^4^ /mL). The initial RHLC solution (10 mg/mL) was diluted 2- and 4-fold in culture medium, a blank control group (BC) of fresh culture medium was included and the collagen treatment (CA) groups contained 0–10 mg/mL RHLC or the collagen sponge. The thiazolyl blue tetrazolium bromide (MTT) assay and a microplate reader were employed to measure the OD_490nm_ value, and cytotoxicity was calculated as described previously (He et al. 2020) according to the formula: Relative growth rate (%) = (OD_CA_/OD_BC_) × 100%. The cell morphology of the BC and CA groups was assessed using an inverted optical microscope (XD-202, Jiangnan Corp., Nanjing, China) at 200 × magnification.

#### Film-forming ability of RHLC

RHLC freeze-dried samples and chitosan (CS) powder were weighed and dissolved with stirring in 1% (V/V) acetic acid solution to prepare RHLC and CS concentrations of 1, 1.5, and 2 mg/mL. The different concentrations of RHLC and CS were mixed at a volume ratio of 1:1, stirred and 7 groups, as well as a 1% (V/V) glycerine group, were set up. Then, 20 mL of each solution was poured into 90-mm disposable Petri dishes, and the plates were placed under a draught cupboard and dried naturally for 48 h. The films were peeled from the Petri dishes and observed. Additionally, different concentrations of natural antibacterial ingredients were added to the 1.5% RHLC and 1.5% CS films, including caffeic acid, chrysin, propolis, and puerarin to observe the film-forming ability.

#### Preparation of the RHLC hydrolysate

Freeze-dried RHLC was dissolved in Tris–HCl buffer (pH 8.0) at a concentration of 20 mg/mL. Collagenase (C128711; Aladdin, Shanghai, China) was added to the collagen solution used for the hydrolyzation reaction at 37 °C for 5 h. SDS–PAGE was used to examine the degree of hydrolyzation. The RHLC hydrolysates were transferred to a 100-Da dialysis tube for saline ions and dialyzed using ultrapure water in an ice bath. After dialysis, the RHLC hydrolysed peptides were frozen at − 80 °C.

One mg of the freeze-dried RHLC hydrolysate was weighed in a PE tube and dissolved in 1 mL of ultrapure water. The high-performance gel exclusion chromatography system DAWN HELEOS 8 + (Wyatt Technology Co., Goleta, CA, USA) was used to determine the molecular weight range.

## Results

### Expression of RHLC in *P. pastoris*

**Fig. 1:**
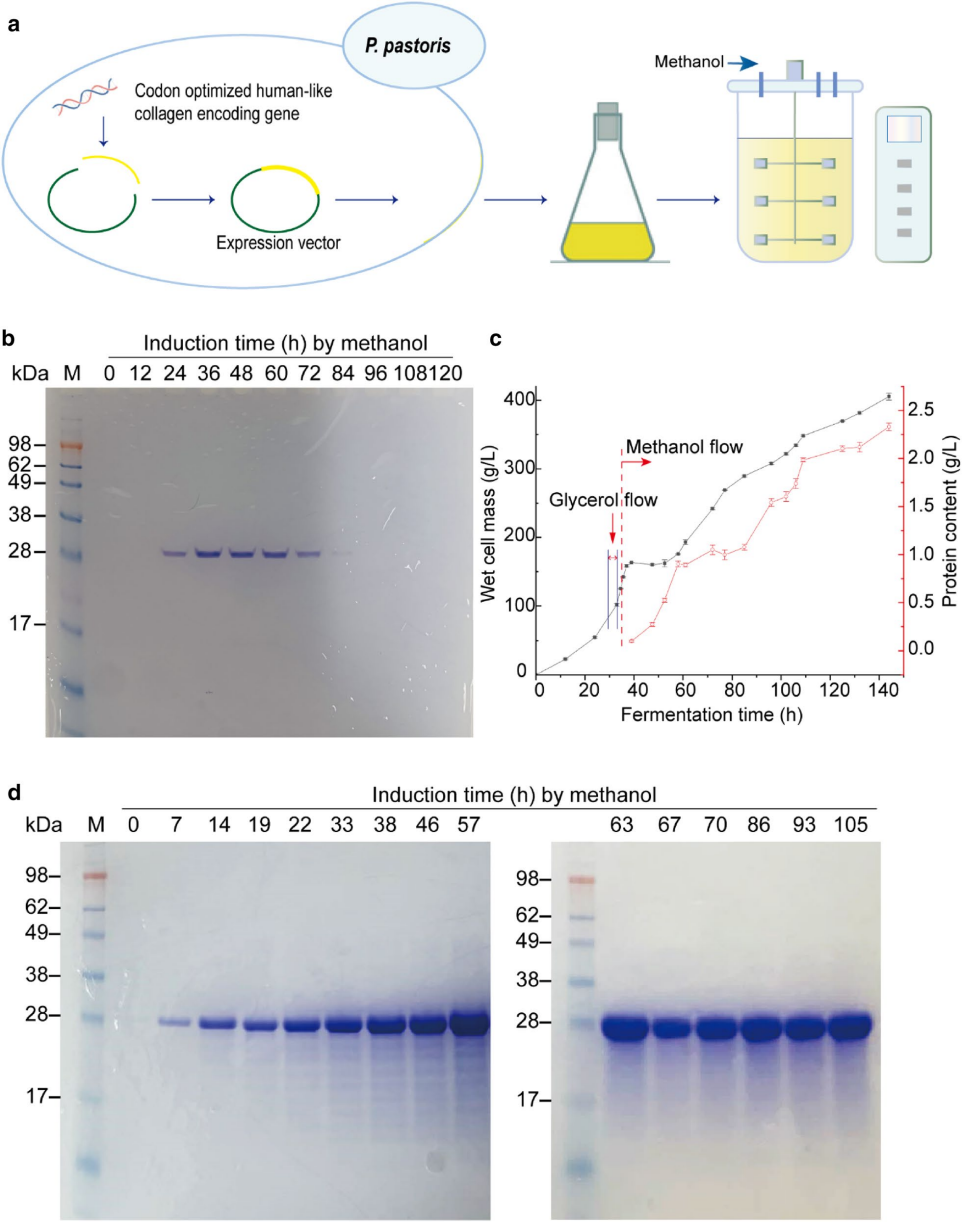
Expression of RHLC via recombinant *P. pastoris*. **a** Sketch map of construction of recombinant *P. pastoris* GS115 with RHLC gene and followed expressed in shake flask and fermenter. **b** SDS–PAGE analysis of RHLC prepared by shake flask fermentation with different induction times and induction by methanol. **c** Wet cell mass (g/L) and protein content (g/L) of RHLC in a 5-L fermenter following induction by methanol. **d** SDS–PAGE analysis of RHLC in a 5-L fermenter with different induction times and induction by methanol

The codon-optimized human-like collagen gene was successfully integrated into the *P. pastoris* GS115 genome, and extracellular secretion was achieved via the α-factor secretion signal. Expression was induced with methanol, and the supernatant of the fermentation broth was subjected to SDS–PAGE to assess collagen expression (Fig. 1a). SDS–PAGE revealed a molecular weight of ~ 27 kDa, which matched the theoretical molecular weight of collagen (27.01 kDa) (https://web.expasy.org/protparam/).

Collagen was first expressed on a small scale in shaker flasks, followed by scale-up fermentation in a 5-L fermenter. RHLC was degraded to varying degrees after several days of shaker flask fermentation (Fig. 1b**)**, and the maximum protein expression was 85–105 mg/L. In contrast, the induced fermentation in the 5-L fermenter performed well in terms of cell mass and protein expression (Fig. 1c and d). The wet cell mass reached 405.3 ± 5.0 g/L during fermentation, consistent with high-density fermentation, and the RHLC expression titre reached 2.33 ± 0.04 g/L, which was ~ 20-fold higher than that achieved by shaker flask fermentation.

### Rapid protein purification

**Fig. 2:**
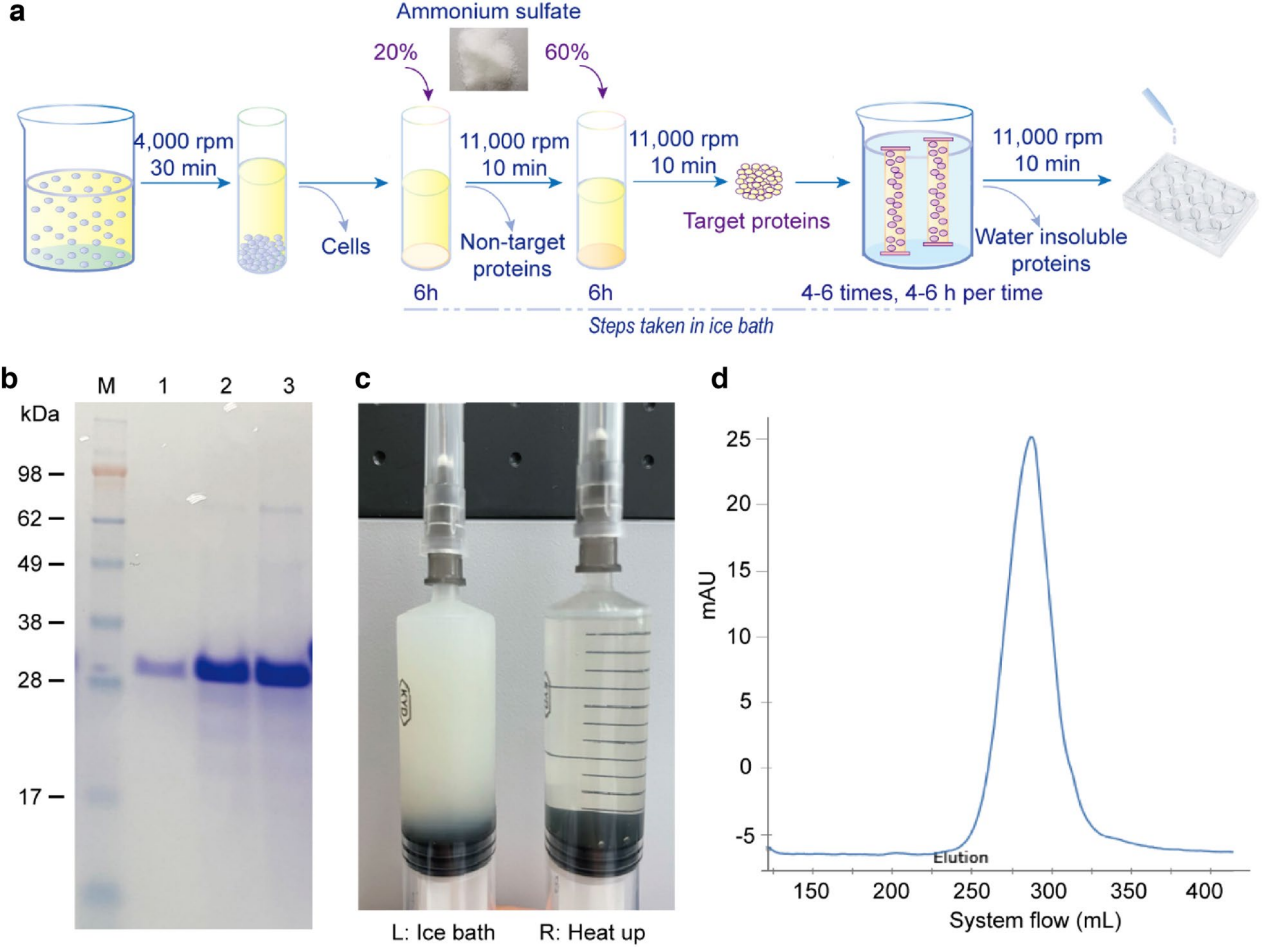
Purification process of RHLC and purity identification. **a** Purification process of RHLC fermentation broth. **b** SDS–PAGE of purified RHLC. Lane 1 was origin fermentation broth, lane 2 was purified RHLC, and lane 3 was redissolved of freeze-dried RHLC. **c** Different states of RHLC at different temperatures (Left was in ice bath and right was at room temperature). **d** Gel filtration analysis of redissolved freeze-dried RHLC samples, with 98% purity

A simple and rapid method is needed to prepare a large number of protein samples, particularly unstable protein samples. In the early stages of this study, column chromatography was used to prepare the samples, but this was slow and not suitable for a large number of samples. Thus, the purified RHLC protein was obtained by ammonium sulphate precipitation combined with dialysis using ultrapure water, and the entire process was carried out in an ice bath (Fig. 2a). During sample preparation, peptides or proteins with molecular weights < 10 kDa were removed by dialysis. Then, RHLC was added to a 12-orifice plate, frozen at − 80 °C and dried in a freeze dryer. The RHLC fermentation broth, the purified samples and redissolved freeze-dried samples, were examined via SDS–PAGE (Fig. 2b) which revealed the high purity of the RHLC.

The RHLC protein was sensitive to temperature (Fig. 1c), and the dialysed sample was gel-like in an ice bath at low light transmittance. This process was reversible. When the sampling process was completed at room temperature or under heat, it immediately became transparent, and it returned to a gel-like state when placed back in the ice bath. It took no more than 48 h from fermentation to freeze-drying, consisting of ammonium sulphate precipitation (6–8 h) twice and dialysis (4–6 times, a total of 24–32 h). The freeze-dried samples were lyophilized for 72 h, dissolved and purity was determined by gel filtration column chromatography (Fig. 2c). The purity of the RHLC was > 98% according to the peak area ratio (Fig. 2d).

### Structural properties and morphological examination of RHLC and the collagen sponge

**Fig. 3:**
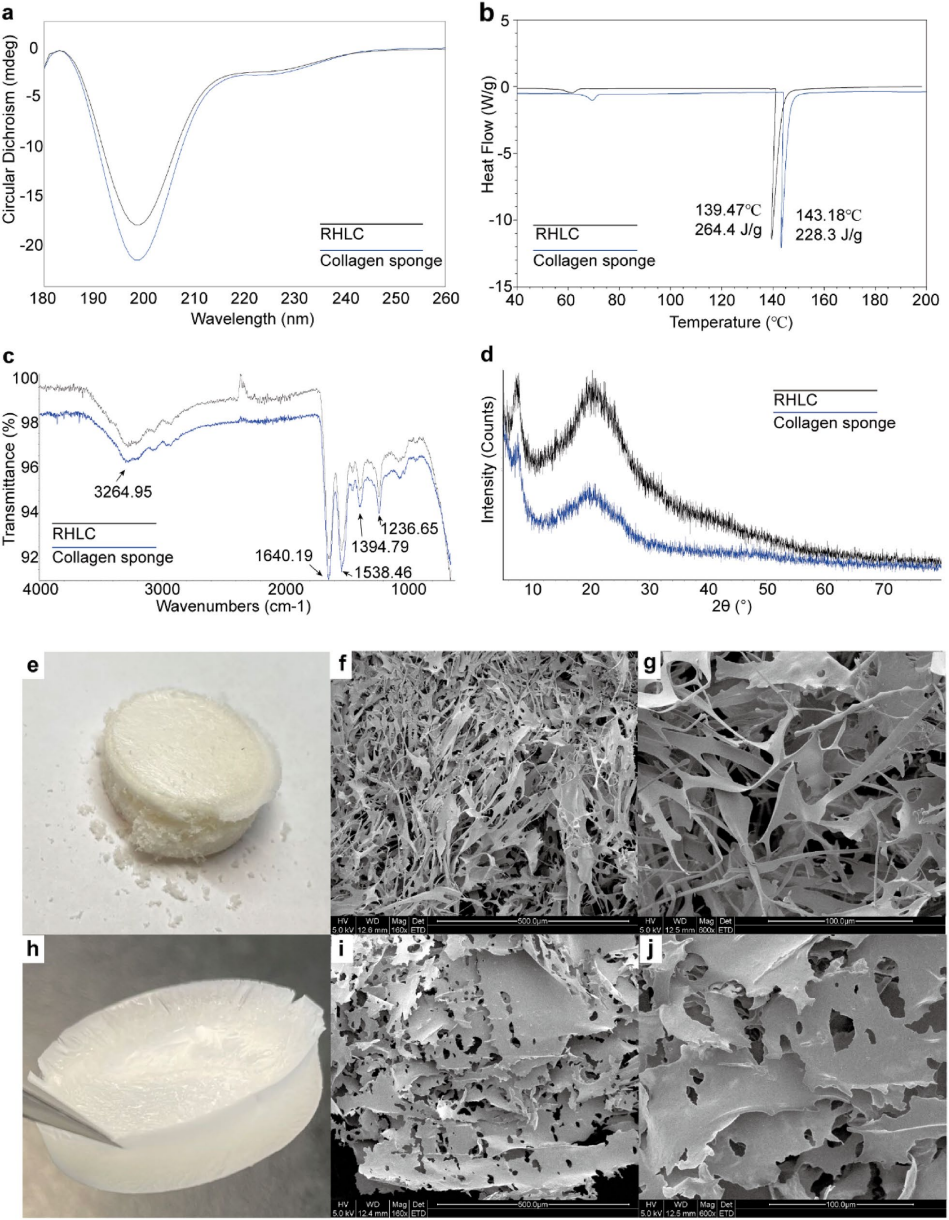
Characteristic of RHLC and collagen sponge samples. **a** Circular dichroism (CD) spectra recorded at a wavelength of 190–260 nm. A significant negative peak at 198 nm indicating a triple helical structure. **b** DSC analysis of the thermal denaturation temperature of freeze-dried samples were heated from 40 °C to 200 °C at a rate of 5 °C/min. **c** Fourier transform infrared spectroscopy (FTIR) analysis at a wavelength range of 650–4000 cm-1 under ATR mode. **d** XRD analysis with the 2θ scanning range was from 5° to 80° with a scan speed of 5° per min. The collagen sponge has a lower 20° (2θ) peak, indicating slight degradation of the triple helical structure. **e**–**j** SEM analysis of freeze-dried RCHL and collagen sponge samples. The three adjacent panels represent the same samples. **e** Freeze-dried RHLC. **f** SEM image at a scale plate of 500 μm. **g** SEM image at a scale plate of 100 μm. **h** Freeze-dried collagen sponge. **i** SEM image at a scale plate of 500 μm. **j** SEM image at a scale plate of 100 μm

The RHLC maintained a triple helical structure. CD and FTIR spectra are useful for studying molecular structure. A minimum CD curve (Fig. 3a) below 200 nm (198 nm in this study) indicated that the RHLC had triple helical characteristics (Salvatore et al. 2020). As the RHLC consisted of a triple-helix structure and did not have a terminal domain similar to extracted collagen, no significant peak appeared at 220 nm that displayed a beta-sheet (Gellermann et al. 2019; Gibney et al. 2021; Zhang et al. 2019). The FTIR results (Fig. 3c) revealed a band typical of collagen molecules, and no obvious differences were detected in the secondary structural content between RHLC and the collagen sponge samples. Briefly, the amide A band, amide II band and amide III bands are the most important absorption peaks corresponding to the secondary structure of collagen. The band at 3264.95 cm^−1^ was a typical amide A band (O–H and N–H vibrations). An amide II band at 1538.46 cm^−1^ corresponded to N–H deformation vibrations and C–N stretching vibrations, while the amide III bands at 1236.65 cm^−1^ corresponded to –CH_2_ stretching vibrations of proline and glycine residues.

Although the molecular structural characteristics of the collagen samples obtained in this study were not significantly different, the collagen sponge experienced slight degradation and destruction of the triple helical structure compared with the original RHLC protein. DSC was used to characterize the thermal stability and structural integrity of the collagen samples (Fig. 3b), and the melting point of the crystalline form displayed a sharp endothermic peak. RHLC had a denaturation temperature and enthalpy of 139.47 °C and 264.4 J/g, respectively. The denaturation temperature of the collagen sponge was 143.18 °C, and enthalpy was 228.3 J/g, which may have occurred due to a change in the integrity of the triple helical structure caused by the formation of the gel (Nöt et al. 2012). The XRD results further confirmed this speculation. A sharp peak was detected at ~ 7–8° (2θ) in the XRD spectrum, indicating aggregation of the microfibrils in the transverse direction, which was also a characteristic absorption peak of the triple helical structure (Fig. 3d). Another broad peak was observed at ~ 20° (2θ), with diffuse reflection caused by multiple structural levels of the collagen microfibrils. The collagen sponge had the lowest two peaks, indicating that the collagen triple helical structure had degraded compared with that of the original RHLC.

The lyophilized RHLC and collagen sponge samples were almost milky-white in colour (Fig. 3e, h). The RHLC was in the microfibril state, and the overall texture was soft and fluffy. However, they were impossible to clamp completely between tweezers due to a lack of rigidity to support their weight. In contrast, the collagen sponge presented a good shape and significant elasticity and could be completely clamped between tweezers. The internal structure observed by SEM provided some support for their appearance. The RHLC had structurally vulnerable fibre edges (Fig. 3f, g) and the collagen sponge presented in a sheet structure. When the RHLC formed the slightly degraded collagen sponge, the fibres aggregated together to form thin sheets with small holes in the middle (Fig. 3i, j). The entire structure of the collagen sponge supported use as a cell scaffold, but any applications will require a cytotoxicity evaluation.

### Potential applications of RHLC

#### In vitro cytotoxicity evaluation

**Fig. 4:**
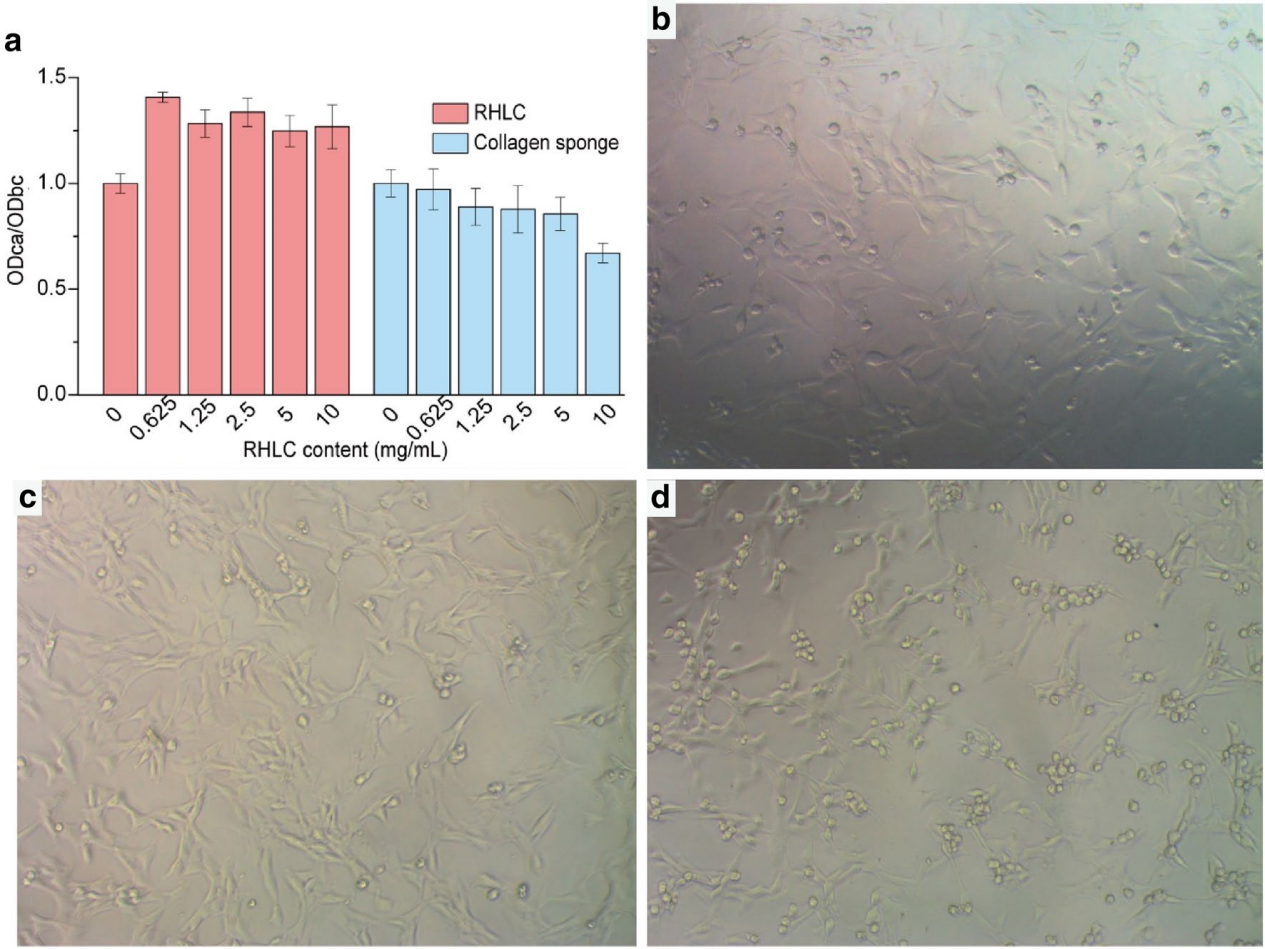
In vitro cytotoxicity evaluation of RHLC (200 ×). **a** OD_CA_/OD_BC_ values of CA groups. **b** BC cells after adding fresh culture medium; **c** CA cells after adding 10 mg/mL RHLC; **d** CA cells after adding 10 mg/mL collagen sponge

To explore the potential applications of RHLC and the collagen sponge in biomedicine, the cytotoxicity of c2c12 mouse muscle stem cells was evaluated using the in vitro MTT assay (Bettini et al. 2015). Collagen samples of different densities were added to the cells, and the OD values were measured to evaluate the effects of collagen on the cells (Fig. 4a). RHLC exerted a growth-promoting effect at all concentrations < 10 mg/mL (OD_CA_/OD_BC_ > 1). Although the collagen sponge had a slight inhibitory effect on cell growth with increasing concentration, it did not significantly inhibit cell growth at concentrations up to 5.0 mg/mL (OD_CA_/OD_BC_ = 0.856), and there was no difference in cell morphology of the collagen sponge (Fig. 4d) compared with the blank control (Fig. 4b) and RHLC (Fig. 4c). Therefore, RHLC and the collagen sponge had good biocompatibility and prospects for applications in biomedicine.

#### Collagen film characteristics

**Fig. 5:**
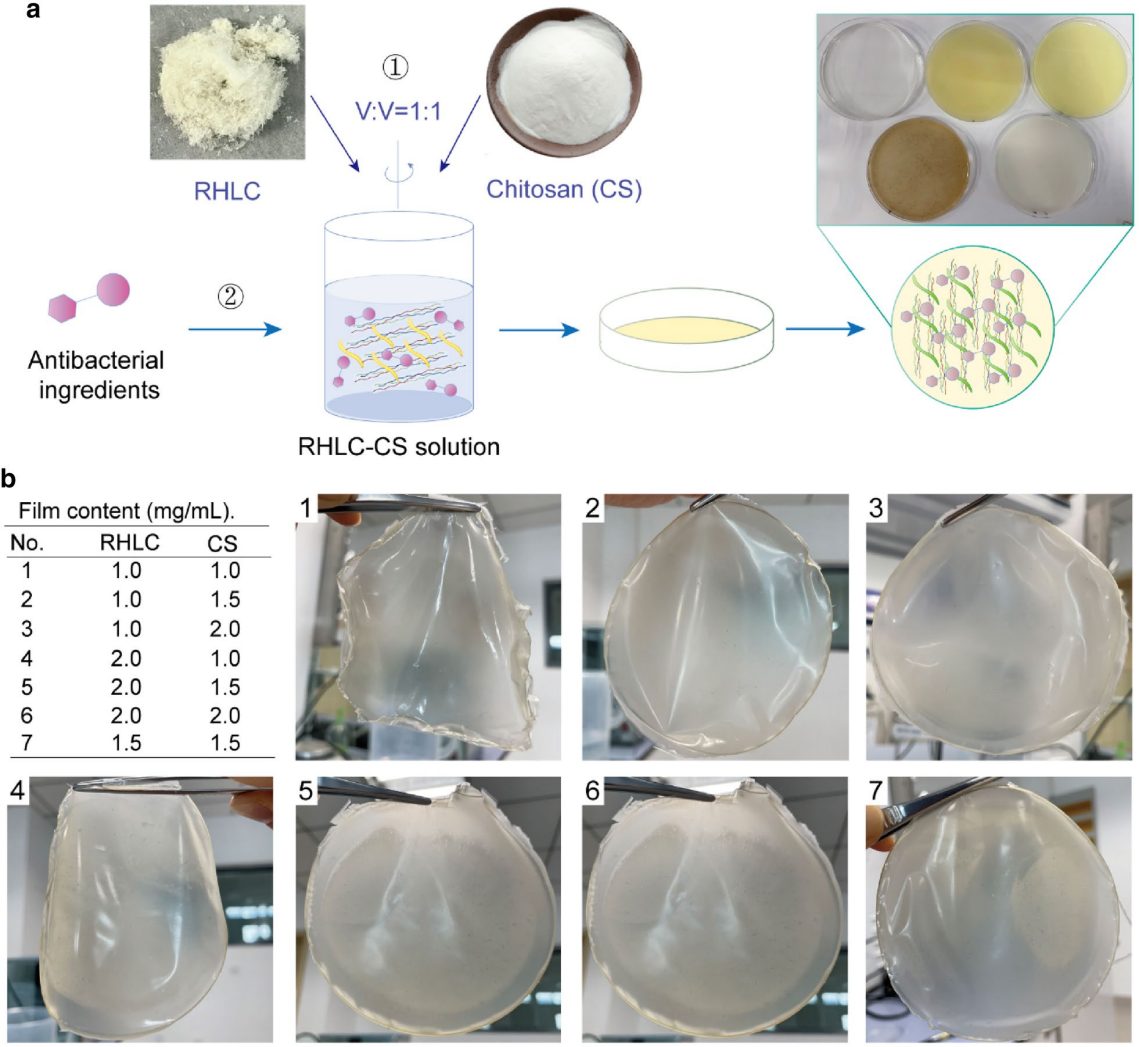
Film-forming ability of RHLC with chitosan. **a** Preparation process of RHLC–chitosan film and additional adding of antibacterial ingredients.** b** Different concentrations and state of RHLC–chitosan (CS) films

CS was chosen to be a component of the collagen film due to its excellent characteristics and broad applications (Andonegi et al. 2020; Heras et al. 2022; Mahendiran et al. 2022; Valenzuela-Rojo et al. 2020) (Fig. 5a). RHLC and CS affected the film characteristics, including thickness, ductility and smoothness of the surface, when they were used in the film. As shown in Fig. 5b, RHLC combined with CS could form a film, and different addition amounts of RHLC and CS are shown in the table. The film thickened and was less soft as CS content increased. However, the RHLC content affected the surface holes in the film. When the RHLC concentration was > 1.5 mg/mL, the film developed holes that decreased the uniformity of the film. Considering the toughness and uniformity of the film material, No. 2 was considered the basal film for further application. Moreover, considering the strength characteristics of the film, more bacteriostatic ingredients could be added to the film to enhance its application value. Different concentrates of caffeic acid, chrysin, propolis, and puerarin were added to the RHLC–CS films and also could form films (Additional file 2).

### Hydrolytic peptides molecular interval distribution

**Fig. 6:**
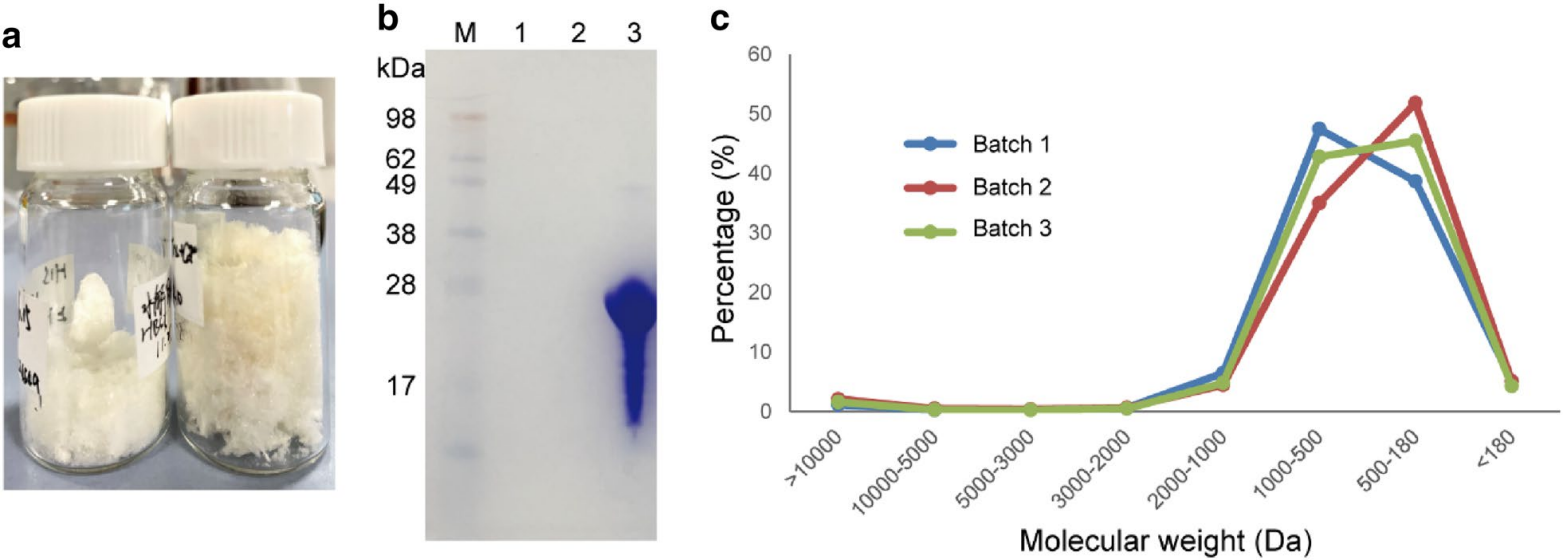
Collagen hydrolysate (peptides) of RHLC and its molecular weight distribution. **a** Freeze-dried samples statement of hydrolytic peptides. **b** SDS–PAGE of collagen hydrolysate of RHLC. Lane 1 and 2 were the production of collagen hydrolysed peptides, line 3 was RHLC before hydrolyzation. **c** Molecular weight distribution of hydrolytic peptides that over 91% were between 180 and 2000 Da. Three batches of hydrolyzation had been examined

The RHLC was completely hydrolyzed by collagenase, then the hydrolytic peptides were freeze-dried after dialysis in ultrapure water with a 100-Da dialysis bag. The lyophilized samples were white thin crispy flakes (Fig. 6a). SDS–PAGE showed the collagen has been completely hydrolyzed and that the collagen lane has been disappeared compared to the initial state (Fig. 6b). The molecular weight range indicated that the collagen hydrolysate was uniform and that more than 91% of different batches were 180AQ6–2000 Da (Fig. 6c).

## Discussion

Collagen is an important functional protein and material in many fields, and its production has gradually shifted from extracting collagen from animal tissue to biotechnology-based microbial cell factory production. In the present study, human-like collagen was expressed in a recombinant *P. pastoris* strain, and the final expression titre of 2.33 g/L was achieved using a 5-L fermenter. This process was coupled with a rapid and easy purification method to obtain a large quantity of the product. A collagen sponge was also prepared from a high-concentration RHLC solution, and its structure and properties were compared with those that had a triple helical structure, a high denaturation temperature and low cell cytotoxicity, indicating promising application potential. The primary potential applications of RHLC were explored which will increase the range of applications.

A high expression titre and an easy purification method are useful for industrial production. Collagen has been previously expressed in *E. coli* BL21 cells at a titre of 300–500 mg/L after culture optimization (Zhang 2017). Herein, we obtained an expression titre of 2.33 ± 0.04 g/L using *P. pastoris* in a 5-L fermenter. *P. pastoris* has also been used as an expression host for collagen production in previous work (Gomes and Salgueiro 2022); however, the expression titre could be improved. First, prolyl 4-hydroxylase tetramer is required to obtain the recombinant collagen (Vuorela et al. 1997). A *P. pastoris* expression system specifically for collagen expression has been developed, human type I–III collagen was co-expressed with prolyl 4-hydroxylase, but the maximum expression titre was 0.6 g/L using a 2-L fermenter (Myllyharju et al. 2000; Nokelainen et al. 2001). The optimized expression conditions and fermentation strategy resulted in a tenfold increase in collagen yield, but the expression titre was low (Ruottinen et al. 2008). The titre was improved to 4.68 g/L for human collagen ɑ1 III (rhCOL3A1) by increasing the fermenter volume to 30 L, but collagen only accounted for ~ 20% of total protein (Li et al. 2015). Therefore, co-expression with prolyl 4-hydroxylase created too complex a collagen expression system and limited the collagen expression titre. In recent years, a recombinant non-hydroxylated gelatine mimetic has been reported in *P. pastoris* and the expression titre was more than 3 g/L (Zhang et al. 2019). We also obtained a recombinant non-hydroxylated human-like collagen from *P. pastoris*, and the expression titre was improved by optimizing the expression conditions and the expression system. In addition, few studies have focussed on the large number of collagen samples needed for industrial production. In the present study, no more than 48 h was required to complete sample preparation because the *P. pastoris* expression system secreted the protein into the fermentation broth (Fig. 1), making it convenient to purify. Our method is more efficient than extracting collagen from animal tissues (Bisht et al. 2021; de Melo Oliveira et al. 2021) or purification by chromatography, making it suitable for large-scale collagen production.

The triple helical and secondary structures of RHLC and the collagen sponge based on CD, FTIR and XRD were analysed in this study. We obtained a basal collagen sponge from a dialysed collagen aqueous solution, which had a spongy texture and was more compact than the original RHLC. Collagen sponges are usually prepared by crosslinking and freeze-drying, which can prolong the degradation of collagen or introduce toxic ingredients (He et al. 2021; Jiang et al. 2017). In the present study, we used a high-concentration collagen liquid with no other components, thereby avoiding these risks. It has been reported that collagen mimetics form based on Pro-Gly-Pro repeats form triple helices at low temperatures and prolonged incubation times (Gellermann et al. 2019). The amino acid sequence of RHLC in this study contained eight G repeat sequences, and it formed a triple helical structure and a gelatinous state following incubation in an ice bath for 10–24 h. The triple helical structure of RHLC makes it a potentially effective biomaterial to support mammalian cells, similar to recombinant human collagen III (Jabaiah et al. 2014). Although peptide bonds rupture and collagen sponges degrade after incubating on ice for hours, this may benefit crosslinking and further absorption by cells because it may facilitate further crosslinking by proteins other than native collagen (Teixeira et al. 2012), which reflects the freeze-dried state and rigid structure of the RHLC gel. Furthermore, when collagen sponges are used as cell scaffolds, collagen variants support cell differentiation (Que et al. 2018). The rapid collagen sponge preparation method developed in this study generated sponges with structural characteristics suitable for biomedical use, but the applications have not been developed.

RHLCs and collagen sponges have high melting temperatures and are non-toxic to cells. Herein, neither the RHLC nor the collagen sponge had a higher melting temperature (> 130 °C) than most extracted collagens (de Melo Oliveira et al. 2021) and some recombinant collagens (Pakkanen et al. 2006). The RHLC and the collagen sponge prepared in this study were also non-toxic to cells. Thus, based on physical characteristics and biocompatibility, RHLC has huge application potential in the food, cosmetics and pharmaceutical industries, as well as in novel materials. RHLC has potential applications as a protein substitute for cultured meat (Zhang et al. 2020), as a food additive (Sun et al. 2021), food filler and as a novel food. Collagen sponges could be used as scaffolds in biomedical and tissue engineering (Dong and Lv 2016; Jiang et al. 2021; Weinrich et al. 2020; Williams, 2019), as well as scaffolds for cell culturing meat (Jaques et al. 2021). Moreover, collagens are candidate biomimetic materials (DeFrates et al. 2018; Gaspar-Pintiliescu et al. 2019) due to their self-assembly behaviour and good biocompatibility; graphene oxide/collagen nanocomposite films (Wei et al. 2019; Yue et al. 2021a), nanofibrous scaffolds (Ghorbani et al. 2020) and collagen/aspartic acid nanocomposite fibres (Yue et al. 2021b) have been reported.

Moreover, to further explore the application potential and value of collagen, we investigated the film-forming ability of RHLC and the molecular distribution of its hydrolysed peptides. CS was a component of the film for its excellent film-forming property, biocompatibility, biodegradability and nontoxicity (Jafari Sanjari and Asghari 2016; Salehi et al. 2016). Materials based on collagen and chitosan films have been widely utilized, such as in biomedicine (Andonegi et al. 2020; Bhuimbar et al. 2019), tissue engineering (Becerra et al. 2022; Pandini et al. 2022), food packaging (Ahmad et al. 2016) and the leather industries (Ocak 2021). CS conferred the film-forming ability of RHLC similar to other extracted collagens or recombinant collagens (Andonegi et al. 2020; Bahrami et al. 2020; Lu et al. 2020). Adding an active constituent (such as a bacteriostatic compound) would improve the value and use of the film (Correia et al. 2022). RHLC–CS films with beneficial compounds added would be uniform, complete and elastic films. Collagen can be hydrolysed into low molecular weight peptides rich in glycine, which would make them biologically active (Ahmed et al. 2020). Thus, collagen hydrolytic peptides are an important by-product with many applications in the medical (Feng and Betti 2017), tissue repair (Sivaraman and Shanthi, 2021), beauty and food (Vidal et al. 2022; Zhang et al. 2018) industries. The molecular weight of most of these collagens was less than 2000 Da (Hong et al. 2019). More application or production details and characteristics will be studied in the future.

## Conclusions

In this study, we obtained a recombinant human-like collagen in the recombinant *P. pastoris* GS115 strain. An expression yield of 2.33 g/L RHLC was obtained using a 5-L fermenter. Additionally, a convenient purification method suitable for large-scale sample preparation was developed, which saved time and cost, with potential application potential for industrial collagen production. Furthermore, we obtained a basal collagen sponge by incubating a high-concentration RHLC aqueous solution on ice to form a gel, which had better toughness, a denser structure and superior application value than the original RHLC. The RHLC and collagen sponge possessed a triple helical structure, high-temperature stability and good biocompatibility. RHLC has a huge potential for industrial production and applications in many areas.

### Supplementary Information

**Additional file 1: Figure S1.** Construction of RHLC expression plasmid via pPIC9K.

**Additional file 2. Figure S2.** Films state of RHLC–CS films with bacteriostatic ingredients.

## Data Availability

All data and materials are available as described in the research article and its supporting information document, which will be given access on the journal's website.
