# Supplementary material for: Expression, characterization, and application potentiality evaluation of recombinant human-like collagen in Pichia pastoris
Source: Bioresour Bioprocess. 2022 Nov 17;9(1):119. doi: 10.1186/s40643-022-00606-3 (PMC10992492; doi:10.1186/s40643-022-00606-3)
Supplement: Supplementary file 1 — Additional file 1: Figure S1. Construction of RHLC expression plasmid via pPIC9K. [file 40643_2022_606_MOESM1_ESM.docx]

**Expression, characterization and application potentiality evaluation of recombinant human-like collagen in *Pichia***

Lingling Ma^1,2^, Xiaolin Liang^1,2^, Shiqin Yu^1,2,3,4^, Jingwen Zhou^1,2,3,4^*

^1^ Science Center for Future Foods, Jiangnan University, 1800 Lihu Road, Wuxi, Jiangsu 214122, China;

^2^ Key Laboratory of Industrial Biotechnology, Ministry of Education and School of Biotechnology, Jiangnan University, 1800 Lihu Road, Wuxi, Jiangsu 214122, China;

^3^ Engineering Research Center of Ministry of Education on Food Synthetic Biotechnology, Jiangnan University, 1800 Lihu Road, Wuxi, Jiangsu 214122, China;

^4^ Jiangsu Province Engineering Research Center of Food Synthetic Biotechnology, Jiangnan University, Wuxi 214122, China.

^*^ Correspondence to:

Jingwen Zhou

Science Center for Future Foods, Jiangnan University, 1800 Lihu Rd, Wuxi, Jiangsu 214122, China.

Phone: +86-510-85914371, Fax: +86-510-85914371

E-mail: zhoujw1982@jiangnan.edu.cn

**Additional file 1**

Recombinant human-like collagen DNA sequence and amino acids sequence


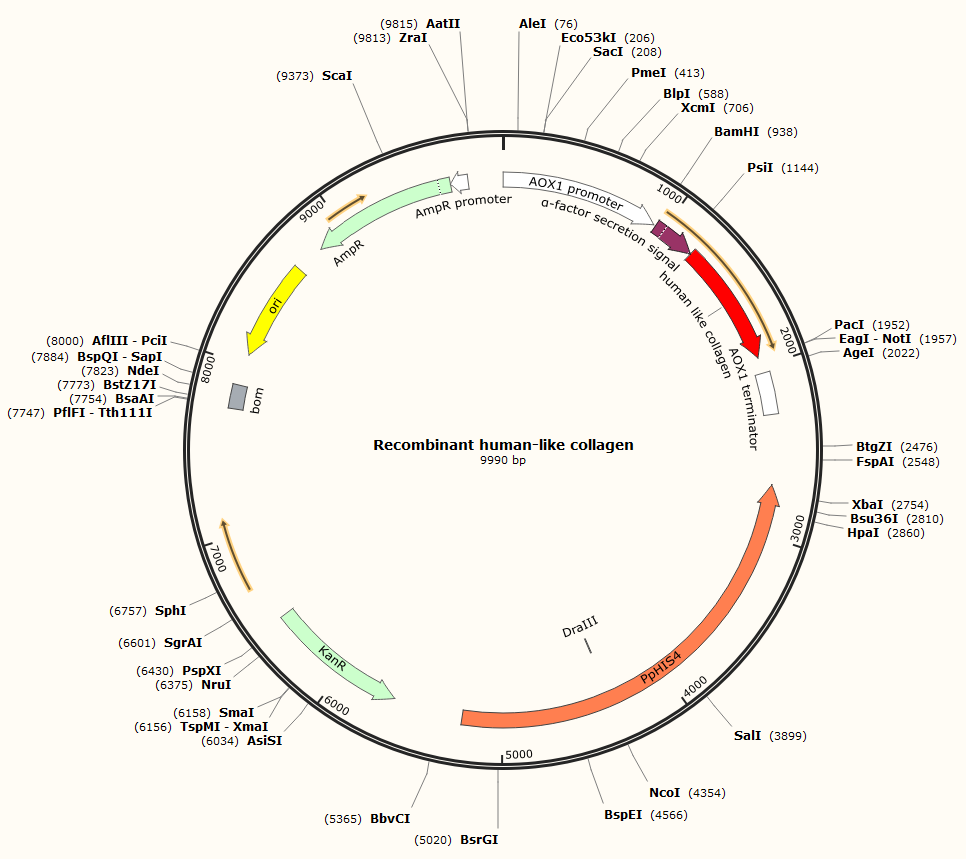


Fig. 1 Construction of RHLC expression plasmid via pPIC9K

Gene sequence of recombinant collagen was as follows.

ATGCATCACCATCACCATCATTTGGTTCCAAGAGGTTCTATGACTTCTGGTGAAAGAGGTGATTTGGGTCCACAAGGTATTGCTGGTCAAAGAGGTGTTGTTGGTGAAAGAGGTGAAAGGGGTGAGAGAGGAGCATCTGGTGAAAGAGGTGATTTGGGTCCACAAGGTATCGCTGGTCAAAGAGGTGTTGTTGGTGAAAGAGGAGAAAGAGGTGAAAGAGGTGCATCTGGTGAGAGAGGAGATTTAGGTCCACAAGGTATCGCTGGTCAAAGAGGTGTCGTTGGTGAAAGAGGTGAAAGGGGAGAAAGGGGTGCTTCTGGAGAAAGAGGAGATTTGGGACCTCAAGGTATCGCTGGTCAAAGAGGTGTCGTTGGTGAGAGAGGTGAAAGGGGTGAGAGAGGTGCTTCTGGTGAAAGAGGTGATTTGGGTCCACAAGGTATTGCTGGTCAAAGAGGTGTTGTCGGTGAGAGAGGTGAAAGAGGAGAGAGAGGTGCTTCTGGTGAAAGAGGTGATTTAGGTCCACAAGGTATTGCTGGTCAAAGAGGAGTTGTTGGAGAAAGAGGTGAAAGAGGTGAAAGAGGTGCTTCTGGTGAAAGAGGTGATTTGGGTCCTCAAGGTATTGCTGGTCAAAGAGGAGTTGTCGGTGAAAGAGGTGAAAGAGGTGAAAGAGGAGCATCTGGAGAGAGAGGTGATTTGGGTCCACAAGGAATTGCTGGACAAAGAGGTGTTGTTGGTGAGAGGGGTGAAAGGGGTGAGAGAGGTGCTTCTTGATTAATTAAGCGGCCGC

Amino acids sequences of RHLC

MHHHHHHLVPRGSMTSGERGDLGPQGIAGQRGVVGERGERGERGASGERGDLGPQGIAGQRGVVGERGERGERGASGERGDLGPQGIAGQRGVVGERGERGERGASGERGDLGPQGIAGQRGVVGERGERGERGASGERGDLGPQGIAGQRGVVGERGERGERGASGERGDLGPQGIAGQRGVVGERGERGERGASGERGDLGPQGIAGQRGVVGERGERGERGASGERGDLGPQGIAGQRGVVGERGERGERGAS
